# A New Approach to Cancer Second Opinions: Overcoming the Challenges of Conventional Oncology Practice by Providing Education to Patients and Physicians

**DOI:** 10.1007/s13187-025-02603-4

**Published:** 2025-03-31

**Authors:** William L. Barrett, Philip D. Leming, Jillian Hunt, Andrew Guinigundo, Lana Uhrig, Robin Zon, Abdul R. Jazieh

**Affiliations:** 1 Department of Radiation Oncology, University of Cincinnati College of Medicine, 3151 Bellevue Avenue, Cincinnati, OH 45219 USA; 2 Cincinnati Cancer Advisors, Cincinnati, OH USA

**Keywords:** Second opinions, Cancer diagnosis, Cancer consultation

## Abstract

Second opinions for cancer diagnosis are frequently sought although they are of variable value to the patient and their primary oncologist. The oncology opinion service launched in 2020 without barriers frequently associated with conventional health systems. Financial barriers were removed; neither patient nor insurance was billed. The service is free of charge. Consult-only service provision, without providing therapy, allows objectivity and minimizes competition between oncology practices. Rapid communication with the treating oncologists allows for consensus treatment planning. Surveys were sent to patients and primary treating physicians assessing the value of the service and to assess the patient’s experience. Between 1/2020 and 12/2023, surveys were sent to patients and their physicians. 539/1244 patients and 186/1319 physicians returned the completed survey. Surveys were scored from 1 to 5 with 5 being the best. Patient surveys rated “perceived value” at 4.95. Physicians scored “helpfulness” at 4.84. Physicians scored 4.07 for the question “enhances your relationship with patient.” This model of providing second opinions offers valuable services to patients and their treating physicians without adding additional financial burden or disturbing their care process.

## Introduction

**“ It is far more important to know what person has a disease than what disease a person has.”**

**Hippocrates**

Seeking a second opinion is common across various illnesses to assure that the patient is receiving the most appropriate treatment. The rate of seeking second opinion varies based on the disease, patient background, and access to healthcare services [1]. For example, approximately 40% of patients with an orthopedic problem seek and receive a second opinion [2–6].

Why is there a need for an additional oncology opinion? The answer is increasing complexity at the same time that the physician and healthcare team have decreasing time to spend per patient. The Human Genome Project completed approximately 20 years ago, accelerated the study of biology, and ignited the whole field of molecular science and medicine. In the biomedical field, over 1 million papers pour into PubMed each year with an 8–9% annual increase over several decades [7]. The first American Society of Clinical Oncology meeting in 1964 had 51 physicians in attendance. In 2024, the total registration was over 45,000. The first scientific meeting in 1965 lasted only 1.5 h compared to the 5 days the current meeting contains. Novel Drug Approvals by the FDA from 2021 to 2023 averaged 47 per year. There are currently over 500,000 trials listed on clinicaltrials.gov. All of the above demonstrates the massive amount of information that needs to be considered when seeing a patient with a new cancer diagnosis and further supports the need for supplemental consultative assistance.

Following an initial cancer diagnosis and treatment recommendation, patients may seek a second opinion for confirmation of diagnosis and treatment plan, expert opinion, and education and advice regarding various approaches to their cancer care journey. Although it has been reported that 15% of patients with newly diagnosed cancer pursue a second opinion [2, 3, 5, 8–11], this rate varies significantly. According to a systematic review, this ranges between 1 and 88% [10]. The majority of patients do not seek a second opinion for multiple reasons, including confidence in their initial recommendation and physician, concern about alienating their primary oncologist, lack of awareness of options for second opinions, challenges associated with expenses, lack of insurance coverage, quality of service variability, lack of communication with the patient’s local physicians and coordination of care, and patient concerns of the second opinion institution requiring the patient to obtain diagnostic evaluation and/or treatment at that site [2, 5, 8, 9].

Furthermore, physicians and the healthcare team are required to dedicate increasing time to various administrative tasks [12, 13] and increasing relative value units (RVUs) [13, 14] by seeing more patients, thereby limiting face time with patients [12, 15]. Ironically, as one study noted, the area that resulted in the highest positive patient satisfaction was how much time a physician spent with a patient [16]. These pressures can lead to dissatisfied patients, suboptimal care, and physician frustration [13–15]. Our goal was to provide an adjunctive service that is entirely patient-focused with close collaboration with the patient’s oncology care team. The education of patients and their oncologists is the primary focus of the service. In this manuscript, we are reporting an overview of our experience and the results of the evaluation surveys completed by the patients and their physicians.

## Methods and Materials

In 2015, a pilot independent oncology advisory practice started providing second opinions to patients at no charge, in a convenient location, both physically and functionally independent of any healthcare system. The usual administrative tasks such as billing, collections, and peer-to-peer requests were eliminated. The goal was to provide an independent, objective, comprehensive evaluation and recommendation, which was rapidly communicated to the treating physicians. This service was provided initially on weekends, without compensation to the physician. The space and equipment were supported through philanthropy.

In response to the demand for services, a full-time medical oncologist and an oncology nurse practitioner were employed in January 2020. This new model’s typical second opinion evaluation includes a thorough review of the patient’s records, spending an average of 2 h face-to-face time with the patient to conduct a comprehensive history and physical examination and extensive patient counseling on various aspects of the disease management and related challenges. The team conducts advanced searches for suitable clinical trials and contacts national and international experts for advice as necessary. Assistance in addressing emotional, social, and financial challenges is provided as needed utilizing community partners and available resources. Prompt communication with the treating oncologist is a priority. In February 2021, a second medical oncologist was added with an additional nurse and medical assistant. This program now employs an executive business director, staff assistant, IT, and marketing team. The program is supported exclusively by an independent 501c3 foundation and remains independent of any healthcare system. Eligible patients reside within a twenty-five-mile radius of the second opinion office. The goal is to raise the level of cancer care throughout the region through patient and physician education.

With increasing requests for services coupled with the complexities of cancer treatments, a second nurse practitioner and third medical oncologist joined and now the program has sub-specialty level expertise in breast, lung, gastrointestinal cancers, melanoma, genomics, and genetics. A dedicated effort is made to include serving underserved populations who might not otherwise seek a second opinion and provide connections to services to assist with drug procurement, financial assistance, mental health services, and other social needs for daily living. A full-time marketing expert is employed to facilitate patient and physician awareness of the service. A full-time community advocate is employed with a focus on recruiting underserved members of the community for education and advice about their disease.

Philanthropic support comes from satisfied patients, local grant foundations, and community members who appreciate the value of the service. Marketing activities have included educational podcasts, special events, media opportunities, and community engagement health fairs.

### Performance Evaluation

To measure the effectiveness of this service, a post-visit paper survey along with a self-addressed stamped envelope is mailed, and an electronic survey is emailed to patients and their treating physicians to obtain feedback about the experience. The survey uses a 1–5 Likert scale, where 1 equates to strongly disagree, 2 to disagree, 3 to neutral, 4 to agree, and 5 to strongly agree. The patient survey includes questions about the overall value of the consultation, the confidence level in treatment recommendations before and after the second opinion visit, and the impact on the relationship between the patient and treating physician (Table 2). The physician survey also focuses on overall value and impact on the doctor/patient relationship but then asks about changes to the treatment plan (Table 3). Descriptive statistics, including mean, were used to analyze all available data.

## Results

The number of patients seen has increased significantly over the ensuing years. In 2020, 229 patients were served. That number more than doubled in 2023 with 500 patients receiving consultations. A wide range of disease sites have been seen, with the most common being melanoma, prostate, breast, colon, and lung cancer (Table 1). These 5 categories represent more than 50% of patients seen yearly.

**Table 1:** Most common diagnoses seen over the last 4 years

	2020	2021	2022	2023
Melanoma	69 (30.1%)	85 (27.9%)	72 (19.1%)	72 (15.3%)
Prostate	21 (9.2%)	29 (9.5%)	49 (13.0%)	58 (12.3%)
Breast	17 (7.4%)	18 (5.9%)	56 (14.9%)	72 (15.3%)
Colon	10 (4.4%)	12 (3.9%)	23 (6.1%)	46 (9.7%)
Lung	6 (2.6%)	20 (6.6%)	21 (5.9%)	31 (6.6%)
Total*	229	305	376	500

^*^Total number of patients seen including all diagnoses

Between 1/2020 and 12/2023, 1244 surveys were sent to the patients and 1319 were sent to the physicians. Five hundred thirty-nine patients returned the completed survey (response rate 43%), and 186 physicians returned the completed survey (response rate of 14%). Physicians receiving multiple surveys when a number of their patients were seen, typically only return the first survey as they consider the quality of the consultation to be consistent across patients. Some patients have surveys sent to multiple physicians (surgical oncologist, medical oncologist, radiation oncologist, primary care). These factors contribute to the percentage of total surveys returned. Press Ganey patient satisfaction survey return rates in the literature range from 16.5 to 33% [17, 18].

The responses are universally highly favorable (Table 2), and most patients rate this as the best medical service they have received in any setting. Surveys sent to physicians revealed the majority of physicians have found the consultation helpful to themselves and their patients and did not feel threatened by the service. The majority of surveys have indicated the experience enhanced the relationship between the patient and the original treating physician (Table 3) while providing relevant education to both the patients and physicians.

**Table 2:** Patients’ ratings of service (*N* = 539)

Question	Mean	Scale
Overall value of consultation	4.95	1 (least) to 5 (most)
Overall confidence in Cincinnati Cancer Advisors recommendations	4.97	1 (least) to 5 (most)
How different was Cincinnati Cancer Advisors recommendation vs. original recommendation?	2.36	1 (not at all) to 5 (very different)
My level of confidence in recommendations I received prior to my Cincinnati Cancer Advisors visit	4.20	1 (low) to 5 (high)
My level of confidence in recommendations I received following my Cincinnati Cancer Advisors visit	4.71	1 (low) to 5 (high)
Likelihood I would recommend CCA to others	4.95	1 (poor) to 5 (excellent)
Timeline — requesting vs. receiving consultation	4.97	1 (poor) to 5 (excellent)
My level of confidence in my current plan of care	4.77	1 (poor) to 5 (excellent)
Has this consultation strengthened your relationship with your treating physician?	4.15	1 (strongly disagree) to 5 (strongly agree)

**Table 3:** Physicians’ ratings of service (*N* = 186)

Question	Mean	Scale
Your impression of the value of the Cincinnati Cancer Advisors consultation to your patient	4.84	1 (least) to 5 (most)
Value of the Cincinnati Cancer Advisors consultation to you	4 78	1 (least) to 5 (most)
How likely are you to refer a patient to Cincinnati Cancer Advisors?	4.69	1 (not likely) to 5 (very likely)
Your perception of the likely value of Cincinnati Cancer Advisors to the Greater Cincinnati community	4.82	1 (not valuable at all) to 5 (very valuable)
Has Cincinnati Cancer Advisors changed your relationship with your patient?	4.07	1 (worsened), 3 (no change), 5 (significantly improved)
Did your patient’s consultation with Cincinnati Cancer Advisors change the treatment plan for your patient?	3.79	1 (not at all) to 5 (very much)
Was any change in the treatment plan as a result of the consultation with Cincinnati Cancer Advisors for the better in your opinion or for the worse?	4.97	1 (much worse), 3 (no change), 5 (much better)
Was the consultation with Cincinnati Cancer Advisors reassuring to your patient?	4.74	1 (not at all) to 5 (very reassuring)
Was the consultation with Cincinnati Cancer Advisors reassuring to you?	4.09	1 (not at all) to 5 (very reassuring)

Recommendations from the second opinion service were often identical or very similar to the original recommendation with 67.8% of the original plans not changing and the benefit of this service being additional reassurance and education for the patient and their families. The second opinion recommendation varied mildly or moderately in 23.5% with original plans being modified to include recommendations from the second opinion program’s physicians, and 1.5% of second opinions differed drastically from the original treatment plans resulting in the patients receiving a completely different treatment recommendation from the original. Treatment plan modifications were unknown for 7.2% of patients. Rapid and informative communication with the treating oncologist is a priority. Incorporation of the second opinion recommendation is at the discretion of the treating oncologist. The second opinion oncologists are regarded as highly experienced, respected physicians with outstanding communication skills, and have longstanding experience in patient and physician education. An annual Best of ASCO conference is hosted by the entity which attracts physicians, nurse educators, and nurses from throughout the region to hear from invited experts.

## Discussion

Second-opinion services for cancer diagnosis are widely available but almost universally associated with patient recruitment for clinical revenue and/or are expensive for the patient. This second opinion service is unique in its purely altruistic mission, independence from current health care constraints, objectivity in being consultation only without providing clinical care, and being without financial hurdles for patients as there is no charge for the service.

The service is potentially helpful in at least six ways: first, it benefits patients by optimizing their treatment plan when appropriate while providing education and reassurance to the patients and family. Second, it assists the treating oncologist in the care of their patients. Third, any new knowledge gained from the consultation serves to educate the oncology team for future patients they may see with similar diagnosis. Fourth, the local oncology team knowing their work may be reviewed by an independent, objective entity, may be motivated to elevate their own performance. Fifth, the team frequently coordinates care by enhancing the communication between multiple oncologists involved in a patient’s treatment, and sixth, this program is able to connect patients to supportive services not known or available to the primary oncologist.

Removing the financial barriers is a critical feature of this program’s services from both the patient’s standpoint and the practitioner’s standpoint. Financial cost was reported as a barrier for seeking a second opinion by patients with cancer [19] and, therefore, eliminating this barrier would encourage more people to pursue a second opinion irrespective of their financial status or insurance provider. For the consultants, removing the financial pressure from the practice equation helps the physician dedicate more time to patient-centered activities rather than focusing on prior authorization or meeting an RVU benchmark.

Patients’ concerns about disturbing the relationship with the primary oncologists have been reported as another barrier of seeking a second opinion [20]. The second opinion experience surveys revealed that both the patients and the oncologists felt that the relationship between them improved after the consultation. The reason for that is the provision of objective opinion with no conflict of interest to the patient and providing timely efficient communication with the primary providers.

This second opinion program provides a medical oasis for its employed physicians and nurses to practice in an environment where well-considered recommendations are the only priority without the administrative and bureaucratic distractions that are such a large part of modern American medicine. The primary objective is for patients to have an excellent plan of care, as great care begins with a great plan. The secondary objective is to enhance the performance of local oncologists. Imparting a different recommendation without alienating the treating oncologist requires finesse and communication skills.

The sustainability of the program currently depends exclusively upon philanthropic support. Corporate support for providing recommendations for employees is an area being considered. Health system support may be possible but only with the ability to maintain complete objectivity. Other revenue-generating approaches may be considered but only if they are consistent with the altruistic philosophy of the service.

This program’s ultimate goal is to minimize the suffering and mortality associated with cancer in the region. We believe this service is very helpful in this regard and will be further beneficial with growth. We are devising additional measures of the benefits of this service. There are currently approximately 11,000 patients diagnosed with cancer in the region, and approximately 1650 would historically (using 15%) be expected to seek a second opinion. This program plans to grow to accommodate any patient desiring this service. The scalability of the concept is under review, and a second office has been recently opened in another city with identical format and service.

Even when the recommendations do not differ from the plan of care of the primary oncologist, the service provides reassurance and education. The team takes more time with the patient than their treating oncologist who is typically under pressure to see many patients in a short period of time. Obtaining more information and education has been reported as a major expectation of patients and may help in reducing uncertainty. Oncologists may overlook this as a reason for a second opinion [21]. The recommendations are communicated to the treating oncologist and the patient. Patients are consistently more confident in their plan of care as a result of the second opinion, often improving adherence with the recommendations of the original oncologist. The education provided to both patients and their oncologists is highly valuable to each.

## Summary

In summary, this new second opinion program provides a unique service to patients with cancer in the region. It has been received favorably by the patients and their oncologists. Future expansion of the services by adding more consultants and serving different geographical locations is underway. This model could be emulated by others in different geographic regions with the prerequisites of (1) a high level of medical expertise, (2) a highly supportive and well-organized structural support system to provide the service in efficient and beneficial fashion, and (3) significant philanthropic support.

A limitation of the study is the percentage of surveys returned. The 43% of surveys returned by patients and the 14% by physicians represent a minority but are consistent with expected survey return rates in the literature [17, 18]. Another limitation is the uncertainty of the impact of the consultation relative to survival, toxicity, quality of life, and expense. We are in the process of developing a method for longitudinal evaluation to meet this need for assessment.

## Data Availability

Data available upon request
